# Combination of natural polyanions and polycations based on interfacial complexation for multi-functionalization of wound dressings

**DOI:** 10.3389/fbioe.2022.1006584

**Published:** 2022-09-09

**Authors:** Shuyang Li, Liya Wang, Jue Zhang, Zijun Zhao, Weifeng Yu, Zhi Tan, Po Gao, Xingtao Chen

**Affiliations:** ^1^ Sichuan Provincial Laboratory of Orthopaedic Engineering, The Aﬃliated Hospital of Southwest Medical University, Southwest Medical University, Luzhou, China; ^2^ Department of Gynecologic Oncology, International Peace Maternity and Child Health Hospital, Shanghai Jiao Tong University School of Medicine, Shanghai Municipal Key Clinical Specialty, Shanghai Key Laboratory of Embryo Original Disease, Shanghai, China; ^3^ School of Stomatology, Wannan Medical College, Wuhu, China; ^4^ Department of Anesthesiology, Renji Hospital, Shanghai Jiao Tong University School of Medicine, Shanghai, China; ^5^ Chengdu Customs Technology Center, Chengdu, China

**Keywords:** interfacial complexation, quaternized chitosan, wound dressing, functionalization, multilayer film

## Abstract

Multi-functionalization of wound dressings with natural polymers is meaningful and remains a challenge. The combination of natural polyanions and polycations appears to be a promising strategy. Still, its performances based on current layer-by-layer self-assembly or homogeneous complexation are mutable and limited. Herein, Ca^2+^-incorporated carboxymethyl cellulose (Ca/Na-CMC) and hydroxypropyltrimethyl ammonium chloride chitosan (HACC) are adopted as the model polyanion and polycation, respectively, to develop multi-functionalized dressings based on interfacial complexation. The dressings exhibit a multilayer structure composed of a polyanion layer (Ca/Na-CMC) for hemostasis and promotion of cell proliferation, a formed polyelectrolyte complex (PEC) layer for structural stability, and a polycation layer (HACC) for antibiosis. Compared to the dressing based on homogeneous complexation, the multilayer dressings show stronger moisture penetrability (around 1,150 g/m^2^/24 h), higher hemostatic activity, and higher antibacterial rate (up to 100%) and promoted effect on cell proliferation. An *in vivo* evaluation using a rat full-thickness skin defect model reveals that the multilayer dressings can accelerate wound healing in 2 weeks. Overall, owing to interfacial complexation resulting in separate layers, the performances of polyanions and polycations after combination are more predictable, and their biological functions can be effectively preserved. These findings not only support the extensive application of multilayer dressings but also offer an alternative strategy for multi-functionalizing wound dressings with natural polyanions and polycations.

## 1 Introduction

Skin is the primary wall of the human body to protect against microorganisms and dehydration and is the most frequently injured organ (Zhao et al., 2017; Qu et al., 2018). It is difficult for injured skin, a wound, to maintain its protective function, and the wound healing process is susceptible to external stimuli (Graca et al., 2020). Hence, the primary function of wound dressings is to protect the wound during its healing process. Unfortunately, concerning the complexity and irregularity of wound healing (Moeini et al., 2020), traditional wound dressings focused on the protective function are insufficient and inefficient in promoting wound healing. The uncontrolled bleeding can seriously obstruct and delay wound healing and even cause mortalities in severe cases stated elsewhere (Li et al., 2020). Additionally, microbial infection is a critical issue responsible for prolonged inflammation and can also be life-threatening (Huang et al., 2020; Rahimi et al., 2020; Xuan et al., 2020); wound dehydration damages the moist microenvironment needed for healing (Varaprasad et al., 2020; Nuutila and Eriksson, 2021). Therefore, wound dressings with multifunction, including hemostatic effect, antimicrobial activity, ability to encourage tissue regeneration, etc., are promising strategies for effective wound management (Yu et al., 2019; Li et al., 2020).

Natural polymers, such as collagen (Lin et al., 2019), alginate (Li et al., 2017), hyaluronic acid (Tang et al., 2020), chitosan (Neto et al., 2019; Yu et al., 2019), and cellulose (Tavakolian et al., 2020), have attracted increasing attention for wound dressing application owing to their ideal biocompatibility, high hydrophilic favorable for absorbing excessive exudate and preventing wound dehydration, hemostatic and antibacterial potentials (Hu and Xu, 2020). In addition, the natural polymers have been extensively investigated for the delivery systems of therapeutic agents (e.g., microRNAs, growth factors) in wound healing (Tang et al., 2020; Deka Dey et al., 2022). These natural polymers and their derivatives are often ionizable under aqueous conditions, resulting in polyanions or polycations, which tend to have different biological functions concerning wound healing. For instance, the polyanions (alginate, hyaluronic acid, etc.) often have hemostatic activity due to their negatively charged surface which is regarded as the vital initiator of activating the intrinsic pathway of the coagulation cascade (Yang et al., 2017; Hickman et al., 2018). The polycations (chitosan, etc.) tend to have antibacterial activity owing to their incorporation with the cytomembrane of bacteria leading to alternative permeability of the cytomembrane (Zhao et al., 2017; Ma et al., 2020; Yin et al., 2021). In this context, combining polyanions and polycations appears to be a promising strategy for the multi-functionalization of wound dressings.

Nevertheless, the performances of the mixture based on current layer-by-layer self-assembly or homogeneous complexation are mutable and limited due to the self-assembly of polyelectrolytes, which has a profound influence on the properties of the compounds (Meka et al., 2017; Chen et al., 2018; Tang et al., 2019). Alternatively, the self-assembly process can be confined to the interface between two oppositely-charged polyelectrolytes, known as interfacial complexation. Because the formed polyelectrolyte complex (PEC) layer *via* interfacial complexation can effectively prevent further complexation and formation of PEC, in the absence of external force (Wan et al., 2016). Thus, the hypothesis is that the construction of a multilayer structure utilizing interfacial complexation is a simple and effective way for multi-functionalization of wound dressings composed of polyanions and polycations *via* maintaining each polyelectrolyte on its corresponding layer.

Carboxymethyl cellulose (CMC) has been developed for wound dressing application due to biocompatibility, biodegradability, high hydrophilic, low cost, and hemostatic activity (Capanema et al., 2018). The hemostatic performance of CMC can be further enhanced by incorporating Ca^2+^ ions, known as Factor IV, involved in the coagulation cascade, using ion exchange (Chen et al., 2015; Kim et al., 2018). Hydroxypropyltrimethyl ammonium chloride chitosan (HACC), a water-soluble chitosan derivative, presents better antibacterial activity than chitosan, which has been extensively applied for wound dressing (Peng et al., 2010; Fan et al., 2015; Rahimi et al., 2019).

Herein, Ca^2+^-incorporated CMC and HACC were adopted as the model polyanion and polycation, respectively, to develop multi-functionalized dressings *via* a one-step sequential casting method based on interfacial complexation, which has not been reported. The composition and structure of the multilayer dressings were investigated and compared with the dressing based on homogeneous complexation. *In vitro* properties including swelling, water vapor transmission, hemostatic and antibacterial activity, and cytocompatibility were investigated. *In vivo* wound healing performance was evaluated in a rat skin defect model. This study aimed to provide a valuable platform to develop multifunctional wound dressings composed of polyanions and polycations *via* interfacial complexation.

## 2 Materials and methods

### 2.1 Materials

Sodium carboxymethyl cellulose (Na-CMC, M_w_ = 2.5 × 10^6^, degree of substitution = 0.9) and calcium chloride (CaCl_2_) were purchased from Aladdin Reagent (China). Hydroxypropyltrimethyl ammonium chloride chitosan (HACC, degree of substitution = 0.9) was obtained from Macklin Reagent (China). All other chemicals were of analytical grade, and used without further purification.

### 2.2 Preparation of multilayer films

Ca/Na-CMC was prepared by a solid-state ion exchange method with some modification (Kim et al., 2018). Briefly, Na-CMC was dispersed in ethanol/water (4/1, v/v) solution containing CaCl_2_ (15% w/v), and stirred for 1 h. Subsequently, the mixture was centrifuged, and the depositions were washed twice with ethanol/water (4/1, v/v) and once with ethanol, sequentially, followed by drying at 60°C overnight.

**FIGURE 1:**
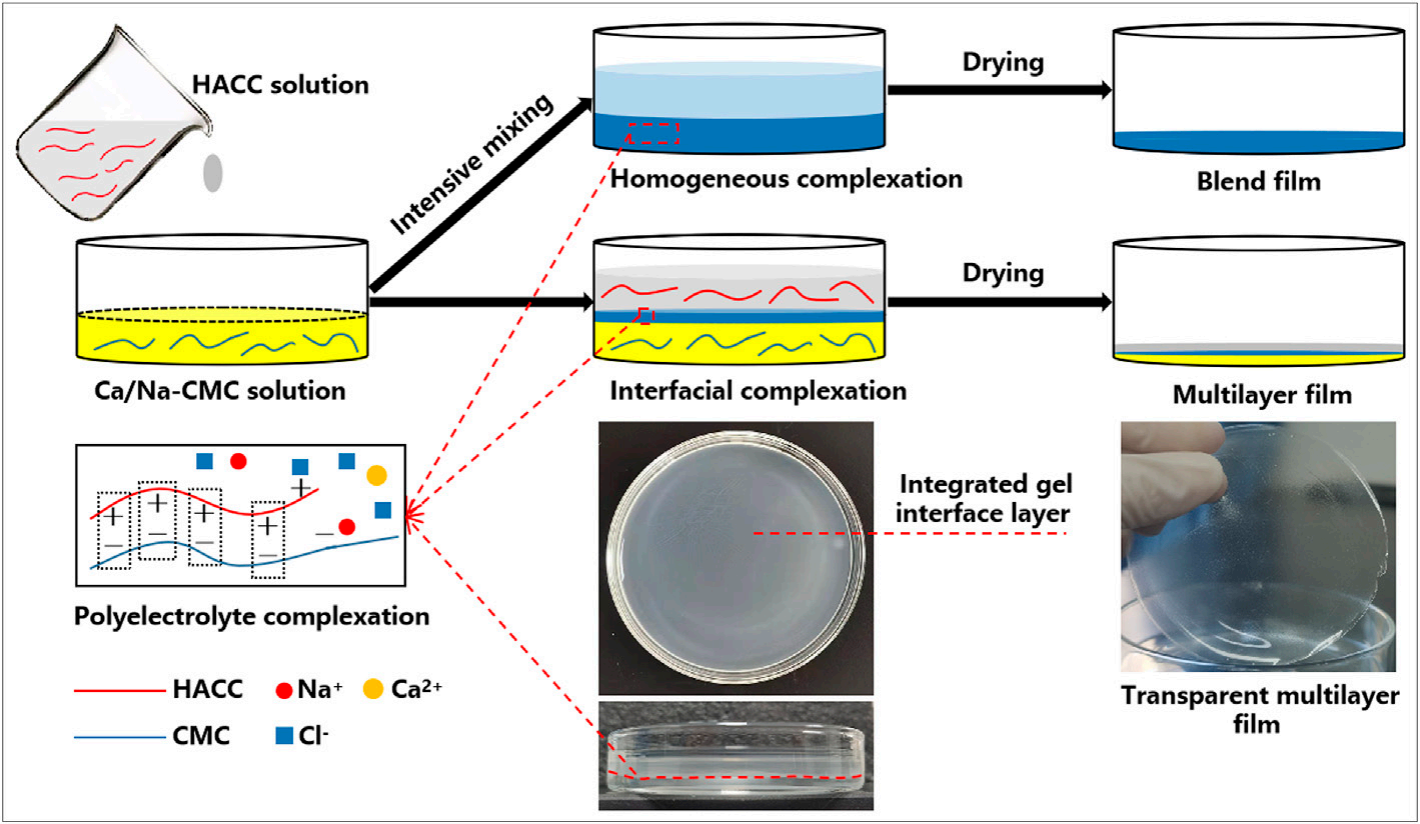
Schematic illustration showing the preparation process of the multilayer and blend films based on interfacial and homogeneous complexation, respectively.

The multilayer films were fabricated through a one-step casting process based on interfacial complexation, as shown in Figure 1. Firstly, Ca/Na-CMC solution (3% w/v) was cast on a glass Petri dish followed by the casting of HACC solution (3% w/v) on the Ca/Na-CMC solution surface. The mixture was held for 30 min while a distinct interface layer was observed between the two solutions due to interfacial complexation. Subsequently, the mixture was dried at 50°C for 48 h, and three multilayer films (MF1, MF2, and MF3) were obtained by varying the weight ratios of HACC to Ca/Na-CMC (1/2, 1/1 and 2/1, correspondingly). The blend films (BF) were prepared by casting the PEC mixture derived from intensive mixing of equal amounts of Ca/Na-CMC and HACC solution (3% w/v) using an emulsifying machine at 12,000 rpm, followed by the same drying process. The Schematics and photographs of the preparation process are presented in Figure 1.

### 2.3 Physicochemical characterization

The films were characterized by Fourier transform infrared spectrometry (FTIR; Nicolet 6,700, Thermo Fisher Scientific, Waltham, MA, United States) and X-ray diffraction (XRD; Shimadzu, Kyoto, Japan). The films’ morphology was analyzed by scanning electron microscope (SEM; S-4800, Hitachi, Tokyo, Japan) after coating with gold in a vacuum. The cross-section was created in liquid nitrogen for SEM observation. Energy dispersive spectroscopy performed a composition assay of the films, Na-CMC, and Ca/Na-CMC powders (EDS; S-4800, Hitachi, Tokyo, Japan). The thickness was measured at five different film sites with a digital micrometer caliper.

### 2.4 Swelling and water vapor transmission

The swelling behavior of the films was investigated in phosphate buffer saline (PBS, pH = 7.4) up to 1 h at room temperature. The dried films (10 mm diameter) were weighed (W_0_) and immersed in PBS. After a predefined time (1, 5, 10, 30, and 60 min), the film was removed from PBS and gently wiped with filter paper to remove surface liquid and weighted (W_s_). The swelling ratio (%) was calculated as (W_s_−W_0_)/W_0_ × 100%.

The film’s water vapor transmission (WVT) was measured according to a standard method (Jantrawut et al., 2019). Briefly, each film specimen was sealed to the open mouth of a 50 ml tube containing 25 ml distilled water. The tube was then incubated in a desiccator at room temperature with 50% relative humidity for 72 h and weighted periodically to construct a cure of weight loss (g) versus elapsed time (h). Based on the slop of the cure, the WVT (g/m^2^/24 h) was calculated as (slop×24)/S, where S (m^2^) is the area of the mouth of the tube.

### 2.5 Whole blood clotting

A whole blood clotting test adopted from previous studies to assess the hemostatic impact of the films was used with some modifications (Chen et al., 2018; Chen et al., 2020). The BF and the multilayer films with different top layers (MF1−MF3 with Ca/Na-CMC top layer, MF2 with HACC top layer, MF2′ composed of the same composition as MF2 except for a Na-CMC top layer) were prepared on the bottom of flat-bottomed vials. Then, citrated blood of Sprague-Dawley (SD) rats (450 μL, blood containing 3.8% (w/v) sodium citrate at a volume ratio of 9:1) was added to the bottom of each vial to submerge the film surface. At the same time, CaCl_2_ solution (45 μL, 0.2 M) was added simultaneously to trigger blood clotting. The vials were incubated with shaking (80 rpm) at 37°C for various times (1, 2, 3, 4, 5 min). Red blood cells (RBCs) not engaged in stable clots were hemolyzed by adding distilled water (15 ml) at each time point. The fluids were transferred into tubes and centrifuged at 1,000 rpm for 2 min. The absorbance of hemoglobin in the supernatant, which represents uncoagulated blood, was measured at 540 nm with a microplate reader.

### 2.6 Antibacterial tests

The antibacterial activity of the films was evaluated by co-cultivation with *Escherichia coli* (*E. coli*, ATCC 8739) and *Staphylococcus aureus* (*S. aureus*, ATCC 6538P) (Huang et al., 2020). Briefly, the film samples (6 mm diameter) were sterilized by the gamma-ray and placed in 24-well plates. The bacterial stock suspensions (1.0 × 10^6^ CFU/ml) were then added to the wells and incubated at 37°C for 2 h. Then, each well, including the film sample, three times was eluted with PBS (pH = 7.0) to detach the bacteria. The eluates were mixed, serially diluted, uniformly plated with plate count agar, and incubated at 37°C for 48 h. The colony on the plates was counted, and the antibacterial rate (%) was calculated as (N_0_−N)/N_0_×100%, where N and N_0_ are the numbers of colonies with and without the films, respectively.

### 2.7 Cell proliferation and morphology

The effect of the films on cell proliferation was performed with L929 fibroblasts using the Cell Counting KIT-8 (CCK-8). The sterilized samples (6 mm diameter) were placed in 24-well plates and immersed in a culture medium (Dulbecco’s Modified Eagle’s Medium supplemented with 10% fetal bovine serum) until cell seeding. L929 cells were seeded at a density of 1×10^6^ cells per well and incubated for up to 6 days at 37°C under 5% CO_2_. At two time points (3 and 6 days), the medium was replaced by a new medium containing 10% CCK-8, and the cells were cultured for 2 h. After being transferred into a 96-well plate, the resulting medium of 100 μL was measured with a microplate reader at 450 nm. The relative growth rate (RGR) was calculated as A/A_0_ × 100%, where A and A_0_ are the resulting absorbances with and without adding to the film samples, respectively.

For observation of cell morphology, cells were spread on a confocal dish containing the film samples and cultured under the same conditions. After 6 days of incubation, the medium containing the films was removed, and the cells were fixed in 4% paraformaldehyde. Before observation by confocal laser scanning microscopy (CLSM; Nikon A1R, Japan), the cells were dark-stained with FITC-phalloidin (Cytoskeleton Inc., America) for 40 min and DAPI (Beyotime Institute of Biotechnology, China) for 10 min, sequentially.

### 2.8 Wound healing in a rat skin full-thickness defect model

The *in vivo* wound healing was evaluated in a rat full-thickness skin defect model (Shi et al., 2019) with the approval of the ethical committee of Southwest Medical University (No. 2020207). All animal experiments strictly abode by the ARRIVE guidelines and the requisitions of the committee. In total, 18 SD male rats (200–260 g, 7 weeks old) were used and kept in line with the guidelines.

All the samples were cut into discs 10 mm in diameter and gamma-ray sterilized. The rats were randomly split into the control group, MF2 group, and BF group. After anesthetization with pentobarbital sodium, two full-thickness wounds (8 mm in diameter) were created on the dorsum of each rat using a medical punch. The samples were applied to the wound and secured using Tegaderm (3 M). At three time points (0, 7, and 14 days), the photographs of the wound area were taken, and the wound closure ratio was calculated as (1−S/S_0_) × 100%, where S and S_0_ are the current and initial areas of the wound, respectively. The rats were euthanized and their skin specimens were collected from the wound sites for histological analysis. Briefly, the specimens were fixed in 10% formalin solution, dehydrated in grading ethanol, embedded in paraffin, sectioned, stained with hematoxylin and eosin (H&E), and observed by a light microscope, sequentially.

### 2.9 Statistical analysis

All the experiments were performed in triplicate or more, and the data points were expressed as mean ± standard deviation (SD). Statistical analysis was performed using one-way ANOVA with post hoc tests, and statistically significant differences are declared when *p* < 0.05.

## 3 Results

### 3.1 Composition, structure and morphology analysis

**FIGURE 2:**
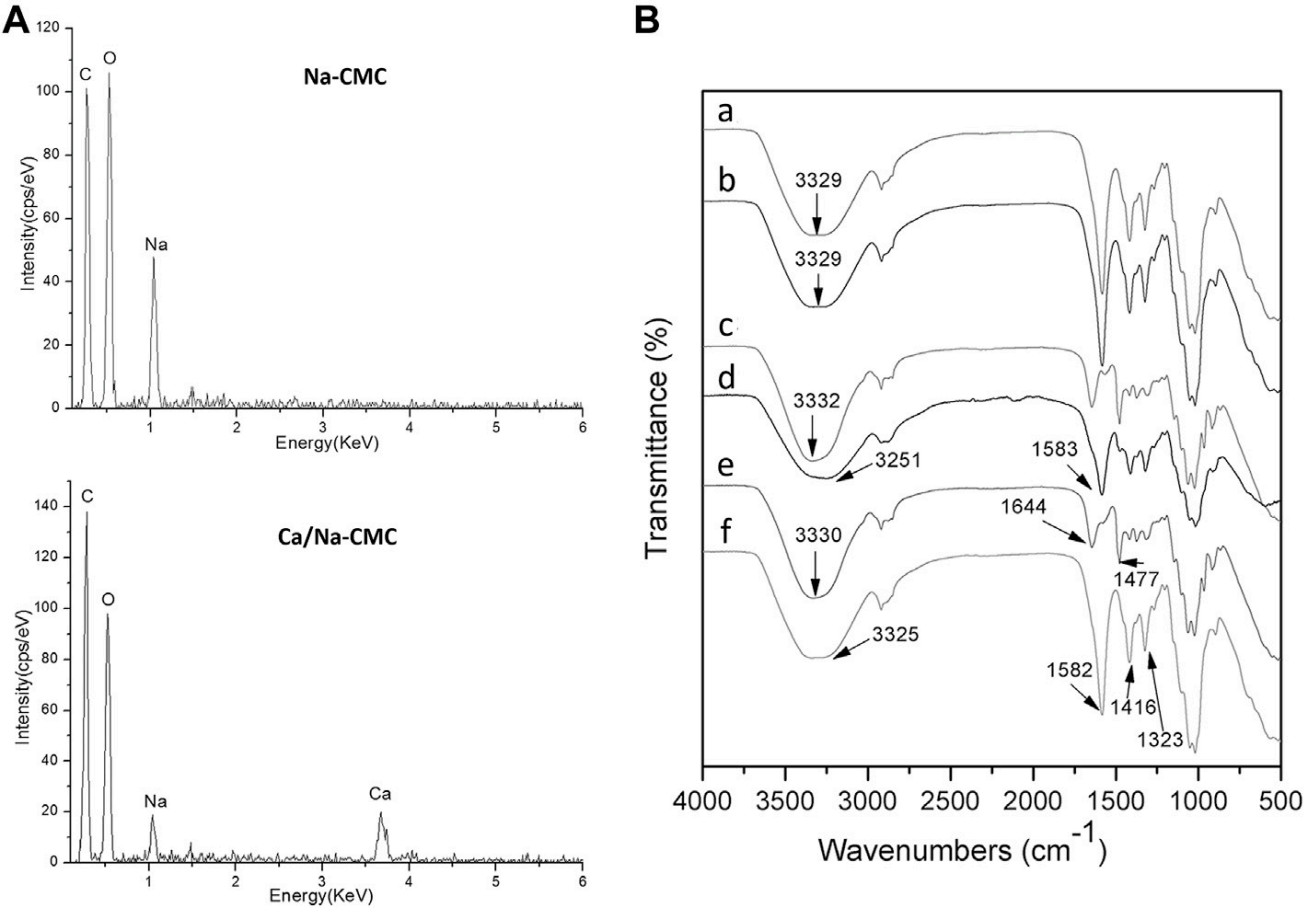
**(A)** EDS spectra of Na-CMC and Ca/Na-CMC. **(B)** FTIR spectra of Na-CMC **(A)**, Ca/Na-CMC **(B)**, HACC **(C)**, BF **(D)**, HACC side **(E)** and Ca/Na-CMC side **(F)** of MF2.

The EDS analysis in Figure 2A demonstrated the presence of Na and Ca in the Ca/Na-CMC, and only Na was presented in the Na-CMC. Additionally, while CaCl_2_ was used as the ion exchange solution, Cl was not detected in Na-CMC and Ca/Na-CMC. Figure 2B represents the FTIR spectra of Na-CMC and Ca/Na-CMC showing the same profile without any shift, suggesting the polymer chain structure remains unchanged. These results demonstrated that the ion exchange proceeded well, partially substituting Na^+^ with Ca^2+^ in the polyelectrolyte.

The FTIR spectra of each side of the multilayer film and the blend film are presented in Figure 2B. For the Ca/Na-CMC side of the multilayer film, the broad band around 3,325 cm^−1^ was assigned to the stretching vibration of O–H, and the–COO^–^groups showed asymmetric stretching vibration at 1,582 cm^−1^ and symmetric stretching vibration at 1,416 and 1,323 cm^−1^. Compared with pure Ca/Na-CMC, the distinct peaks were basically unchanged and no new peak was found. For the HACC side, the broad band around 3,330 cm^−1^ was attributed to O–H and N–H stretching vibration; two peaks at 1,644 and 1,477 cm^−1^ corresponded to C=O stretching of amide I and C–H bending of the trimethylammonium groups, respectively, which was almost the same as pure HACC. The results indicated that the polymer chain characteristics of either Ca/Na-CMC or HACC were preserved on each side. As for the BF, the two peaks corresponding to amide I from HACC disappeared or overlapped with asymmetric stretching vibration from Ca/Na-CMC at 1,583 cm^−1^. The band attributed to O–H and N–H red-shifted to 3,251 cm^−1^, suggesting new intermolecular bonds such as ionic interaction and hydrogen bonding between the two polymers.

**FIGURE 3:**
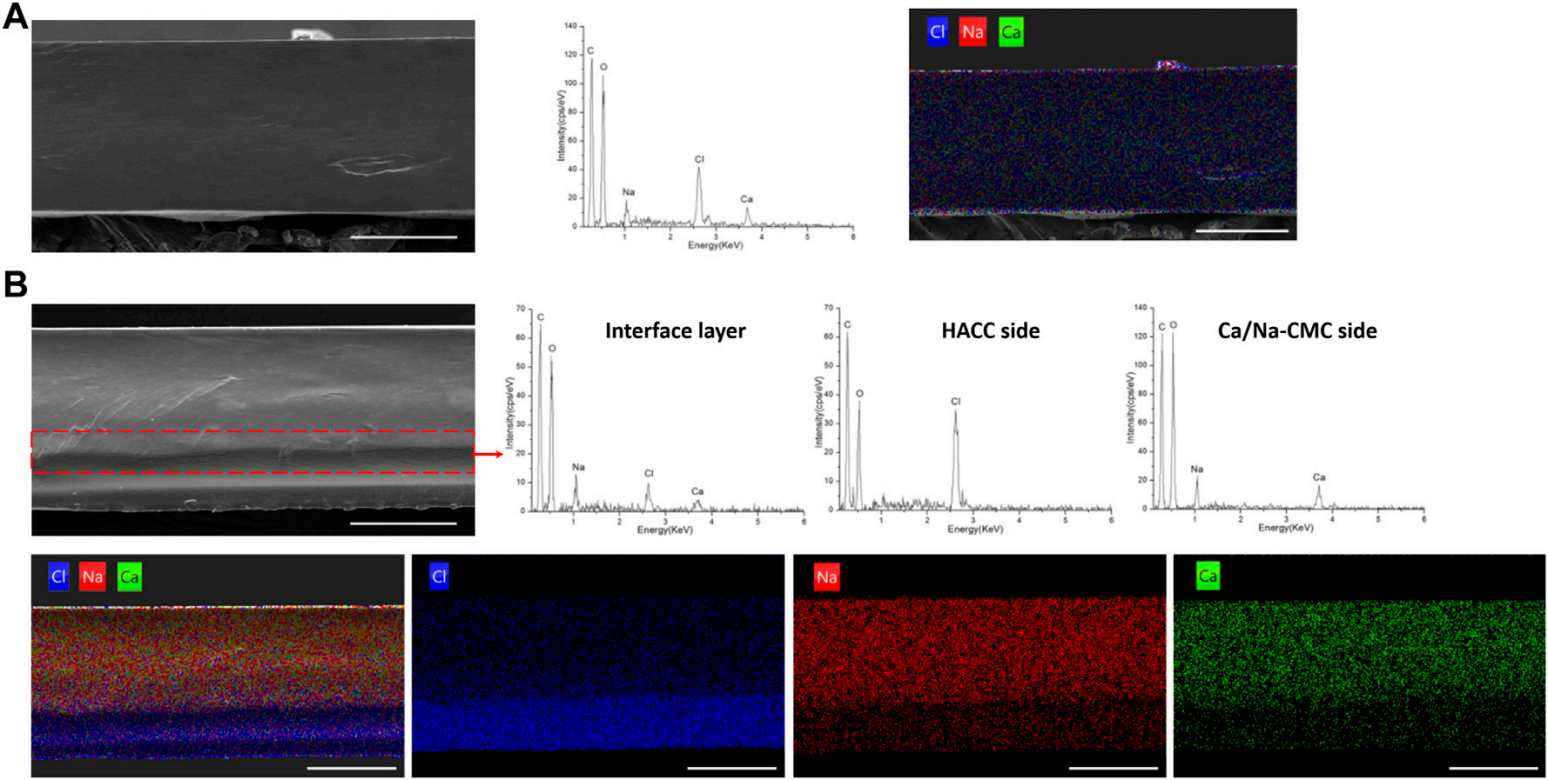
Morphology and composition analysis. **(A)** SEM image of the cross-section of BF, and corresponding EDS spectrum and mapping (chlorine, blue; natrium, red; calcium, green). **(B)** SEM image and corresponding EDS mapping of the cross-section of MF2, and EDS spectrum of the interface layer (in the dotted box), HACC side and Ca/Na-CMC side of MF2. (scale bar: 100 μm)

Figure 3 shows the morphology and elementary composition of the blend and multilayer films. Figure 3A exhibited a homogenous cross-section of the BF, reflecting homogenous complexation. In addition, the EDS spectrum and mapping result confirmed homogenous complexation. Na and Ca from Ca/Na-CMC and Cl from HACC were evenly distributed and accounted for a fair ratio.

In the cross-section image of MF2 in Figure 3B, the observed interface layer is labeled by a dotted box, and the EDS spectrum detected Na, Ca, and Cl. On the other hand, the EDS spectrum of the HACC side established that almost only HACC was presented, since Cl rather than Na or Ca was detected. For the Ca/Na-CMC side, Na and Ca were detected rather than Cl, suggesting the presence of Ca/Na-CMC rather than HACC. Corresponding EDS mapping of the MF2 cross-section showed that the distribution of Cl was uneven and denser close to the HACC side, while Na and Ca were denser close to the Ca/Na-CMC side. Moreover, the two interfaces identified by the SEM image or corresponding EDS mapping were almost coincide, demonstrating the multilayer structure. The results show the homogenous and multilayered microstructures of the blend and multilayer films, respectively, and suggest that the composition of each polyelectrolyte is preserved on its corresponding side of the multilayer film, following the FTIR results.

### 3.2 Swelling behavior and water vapor permeability

**FIGURE 4:**
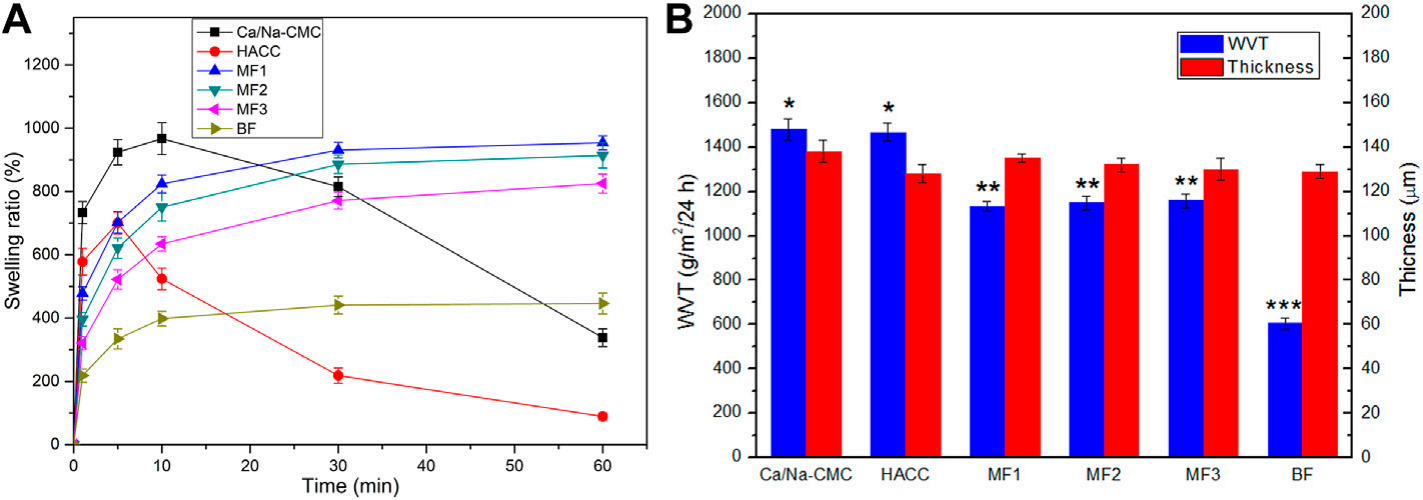
Swelling ratio **(A)**, WVT **(B)** and thickness **(B)** of Ca/Na-CMC film, HACC film, three multilayer films (MF1-3) and BF. *p < 0.05 relative to MF1, MF2 or MF3. **p < 0.05 relative to BF. ***p < 0.05 relative to CMC-Ca or HACC.

Figure 4A represents the films’ swelling ratio as a function of the time in the PBS. The MF1-3 and BF presented a rapid water uptake in the initial ten minutes. The stable equilibrium swelling of MF1, MF2, MF3 and BF, were 954%, 914%, 825%, and 450% respectively. Conversely, pure Ca/Na-CMC and HACC films tended to dissolve the rest of the time. Due to their high solubility dramatically declined the swelling ratio after an initial (five or ten minutes) water uptake phase. The swelling ratio of MF1-3 was significantly higher than that of BF throughout the test. This swelling behavior can be attributed to interfacial complexation resulting in a lower degree of complexation than homogeneous complexation.

The WVT and thickness of the films are shown in Figure 4B. The WVT of MF1-3 (around 1,150 g/m^2^/24 h) was significantly higher than that of BF (605 g/m^2^/24 h) but lower than that of Ca/Na-CMC (1,480 g/m^2^/24 h) and HACC (1,467 g/m^2^/24 h). No significant difference was found between the multilayer films. Considering almost the same film thickness (around 130 μm), these results suggested that the WVT of the films decreased with increasing the degree of polyelectrolyte complexation.

### 3.3 Hemostatic activity *in vitro*

**FIGURE 5:**
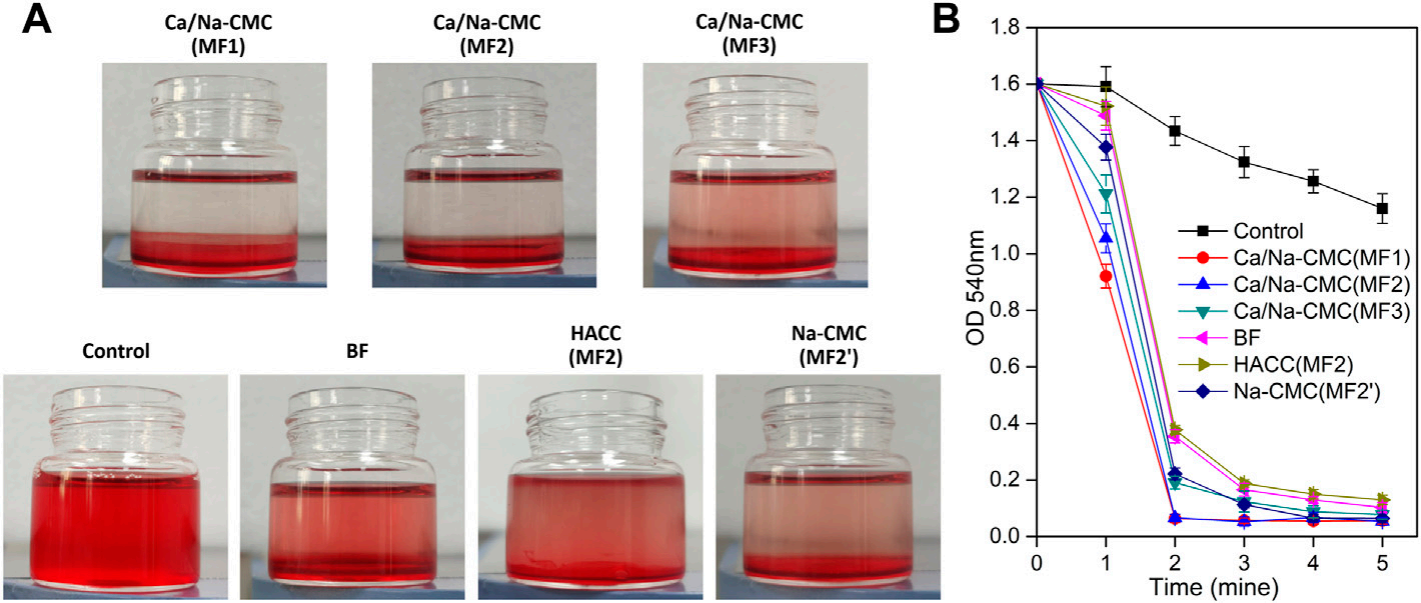
Blood clotting efficiency. **(A)** Photographs of the vials after 2 min clotting with the BF and multilayer films with different top layers: MF1−MF3 with Ca/Na-CMC top layer, MF2 with HACC top layer, MF2′ with Na-CMC top layer. **(B)** Blood clotting efficiency determined by measuring hemoglobin absorbance (540 nm) from RBCs not involved in clots.

The ability of the films to activate and induce blood clotting was investigated through a whole blood clotting test. As presented in Figure 5, the hemoglobin concentrations (absorbance at 540 nm), which corresponded to red blood cells not engaged in clots. MF2 with Ca/Na-CMC top layer was considerably lower than MF2′ with Na-CMC top layer at every time, suggesting that incorporating Ca^2+^ into the multilayer film could improve hemostatic activity.

Furthermore, the lower absorbance for MF2 with Ca/Na-CMC top layer than that for BF indicated that the high hemostatic activity of Ca/Na-CMC was preserved owing to interfacial complexation. In contrast, homogeneous complexation resulted in suppressed hemostatic activity. Interestingly, no significant difference was found between MF1 and MF2 with the same Ca/Na-CMC top layer. However, MF3 showed a significantly higher absorbance even if it also possessed Ca/Na-CMC top layer. A reasonable explanation was that compared with MF1 and MF2, the Ca/Na-CMC layer of MF3 was too thin to sustain blood coagulation. Unsurprisingly, MF2 with HACC top layer exhibited an absorbance much higher than MF2 with Ca/Na-CMC top layer. It was suggested lower hemostatic activity of the HACC layer than the Ca/Na-CMC layer. Therefore, it concluded that Ca/Na-CMC rather than HACC was responsible for promoting the hemostatic activity of the films, and it was more effective as a separate layer rather than forming PEC with HACC.

### 3.4 Antibacterial activity

**FIGURE 6:**
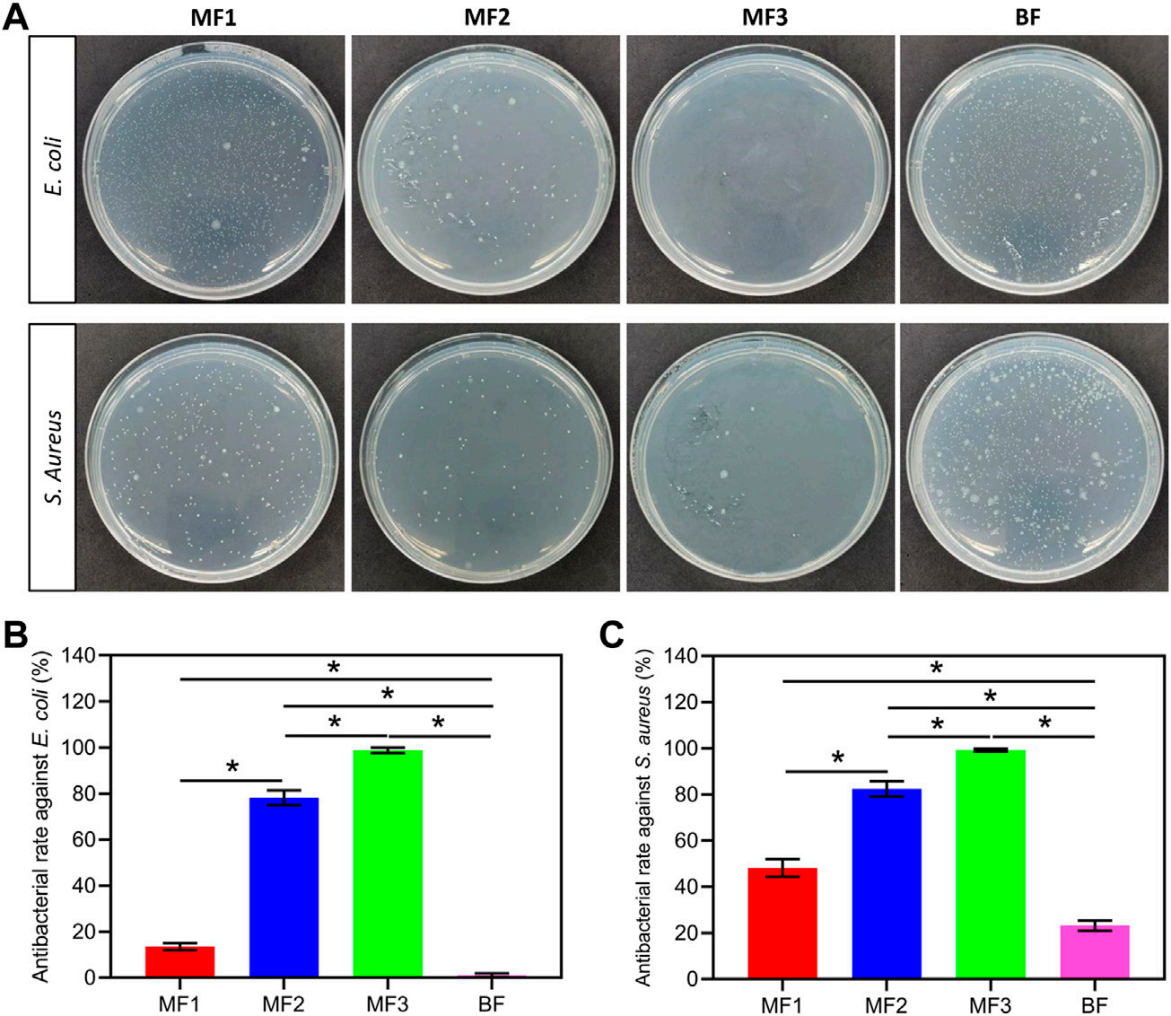
Antibacterial activity of the films. Photographs of plate count agar from different groups **(A)**, and corresponding antibacterial rate against *E. coli* **(B)** and *S. aureus* **(C)**. *p < 0.05.

The antibacterial activity of the films was assessed using *E. coli* and *S. Aureus*, as shown in Figure 6. The pure Ca/Na-CMC film did not perform any apparent antibacterial behavior (data not shown). In contrast, the multilayer films showed a significant increase in antibacterial rate against both *E. coli* and *S. aureus* depending on the ratio of the HACC layer. In particular, the HACC ratio increased 1/3- 2/3, and the antibacterial rate of the multilayer films increased correspondingly from 14% to 99% and 48%–99% against *E. coli* and *S. aureus*. Although the content of HACC in BF was equal to MF2, the antibacterial rate of BF is nearly zero and 23% against *E. coli and S. aureus*, respectively, which is significantly lower than that of MF2. The antibacterial activity of MF1 and BF against *S. aureus* was higher than that against *E. coli*, while the same phenomenon was not evident for MF2 and MF3. These results suggest that HACC can endow the films with antibacterial activity, and it is much more efficient as a separate layer than forming PEC with Ca/Na-CMC.

### 3.5 Cytocompatibility

**FIGURE 7:**
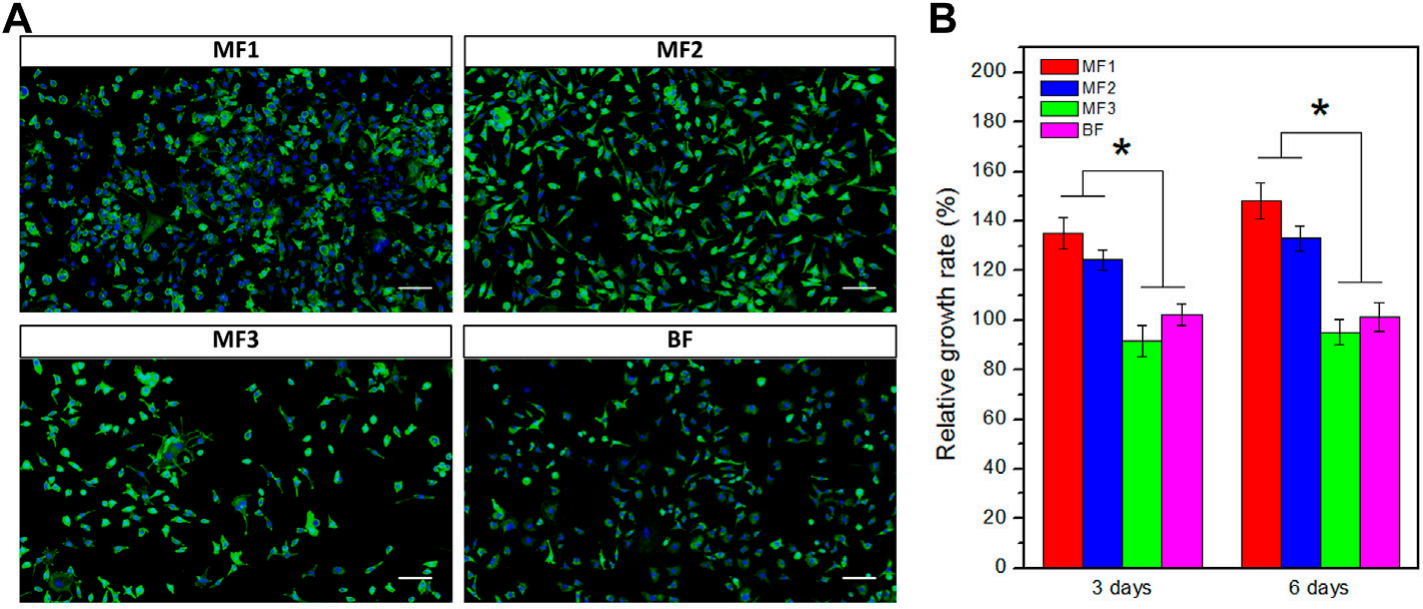
Cytocompatibility evaluation. **(A)** Representative confocal laser scanning microscopy images of L929 cells after 6 days of incubation with different films. Cytoskeleton marker FITC-Phalloidin in green and nucleus marker DAPI in blue (scale bar: 100 μm). **(B)** Cell proliferation data from the CCK-8 assay after 3 or 6 days of incubation. *p < 0.05.

Cytocompatibility of the films was evaluated by CCK-8 and further investigated with the observation of cell morphology by CLSM. Cell proliferation results in Figure 7B revealed that RGRs in all the films were more than 91% after 3 or 6 days of incubation, indicating the lack of cytotoxicity. Moreover, the RGRs in MF1 and MF2 were 135% and 124% after 3 days and slightly increased to 148% and 133% after 6 days, respectively, which were significantly higher than that in MF3 and BF (around 100%), suggesting an enhanced effect of Ca/Na-CMC layer on cell proliferation. It is worth noting that despite sharing the same content of Ca/Na-CMC and HACC, MF2 showed much higher RGR than BF. The results of cell morphology after 6 days of incubation in Figure 7A confirmed cell proliferation results. Cells cultured with all the films spread well and displayed typical fibroblast morphology with shuttle-like or multiple angle shape, indicating good cytocompatibility. Cell numbers in MF1 and MF2 were found to be more than that in MF3 and BF, which agreed with the cell proliferation results.

### 3.6 Wound healing *in vivo*

**FIGURE 8:**
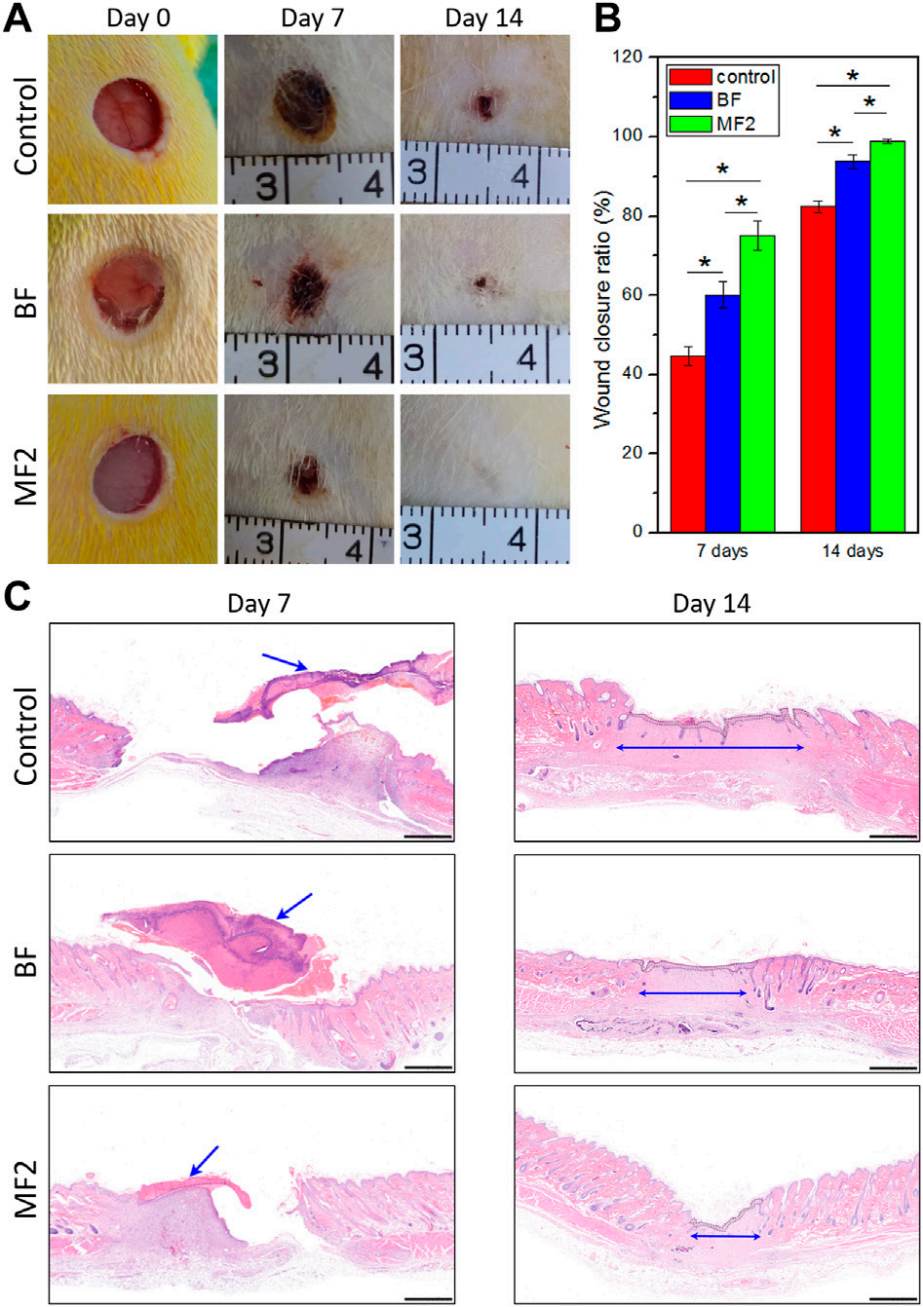
*In vivo* full-thickness wound healing assessment. **(A)** Gross observation photographs of the wound treated with different films for various days. **(B)** Corresponding wound closure ratio. **(C)** H&E staining of the wounds from different groups (scale bar: 1 mm). The arrows indicate blood scabs and the edge of the wounds on days 7 and 14, respectively. The formed epithelium after 14 d of treatment is highlighted with black dotted lines. *p < 0.05.

In order to evaluate the capability of the films to enhance wound healing *in vivo*, MF2 and BF were applied in a rat full-thickness skin defect model. Figure 8A,B depict the wound healing process for up to 14 days. MF2 and BF, attributed to high hydrophilicity, could rapidly adhere to the wound and absorb exudates, including blood, on day 0. After 7 days of treatment, the MF2 group exhibited a significantly higher wound closure ratio than the BF and the control groups. The trend continued after 14 days resulting in almost complete healing with new epidermis for the MF2 group versus 6% and 18% of the unclosed wound for the BF group and the control group, respectively.

The wound healing process was further investigated by histology observation (H&E), as illustrated in Figure 8C. MF2 group showed smaller wound size with less blood scab on day 7, compared to the BF and control groups. On day 14, epithelialization was almost completed in all groups, and the MF2 group showed the minimum unmatured granulation tissue, followed by the BF group and the control group. Consistent with the results of gross observation, MF2 group presented faster and more complete wound closure than the BF group. These results suggest that Ca/Na-CMC/HACC film has an accelerating effect on wound healing, and its performance is better in the form of multilayer film based on interfacial complexation than blend film based on homogeneous complexation.

## 4 Discussion

Wound healing is regarded as a complex and dynamic biological process consisting of four overlying stages: hemostasis, inflammation, epithelialization and remodeling (Homaeigohar and Boccaccini, 2020; Moeini et al., 2020). Regardless of the essential protection function, wound dressings with multifunction pointing at accelerating or promoting any of the four stages of wound healing are particularly useful, and have attracted the interest of a growing number of researchers (Li et al., 2017; Yu et al., 2019; Li et al., 2020). To this end, combining polyanion and polycation which have different biological functions, can be a promising strategy to endow wound dressings with multifunction.

However, polyelectrolyte self-assembly between polyanion and polycation profoundly influences this strategy. Compared with current PEC film based on homogeneous complexation, we presented a novel multilayer film based on interfacial complexation (Figure 1). Interfacial polyelectrolyte complexation (IPC), based on interactions at the interface of oppositely charged polymers, has been applied to fabricate fibers and capsules since 1998 (Wan et al., 2016; Do et al., 2017), but has rarely been used for the construction of multilayer films. During the preparation process, it is worth noting that a distinct and integrated gel layer owing to interfacial complexation can be observed at the interface of the two solutions. The SEM and EDS results (Figure 3), coupled with the FTIR spectra (Figure 2B), further confirmed the multilayer and homogeneous structures of the two films, which lay the foundation of this study. The multilayer films are composed of three layers: HACC, PEC, and Ca/Na-CMC (Figure 3B), derived from HACC solution, interfacial PEC gel, and Ca/Na-CMC solution, respectively (Figure 1).

According to the well-established wet healing theory, wounds can heal 3–5 times faster under a suitable moisture microenvironment than a dehydrated one (Varaprasad et al., 2020). On the other hand, wound exudate, essential for providing the moisture, can be detrimental if excessive (Shi et al., 2019). So wound dressings should play a pivotal role in assuring a moist environment as well as absorbing excessive exudate (Varaprasad et al., 2020). In this regard, the swelling behavior and WVT were adopted to determine the capacity of the films to keep a suitable fluid balance for wound healing (Figure 4). The results suggest that unlike the dissolution tendency of pure Ca/Na-CMC and HACC, the swelling behavior of the multilayer films is more like that of the blend film with a rapid uptake stage (≤10 min) followed by a stable equilibrium stage, owing to interfacial complexation. This swelling behavior and adequate WVT are favorable for absorbing excessive exudate unceasingly. Furthermore, the enhanced water absorption and WVT of the multilayer films are more beneficial to absorbing exudate and keeping the wound moist and breathable than the blend film. Additionally, the interfacial complexation can stabilize and integrate the multilayer films during swelling, which is essential for each layer to perform its function after absorbing blood or exudate *in vivo*.

Rapid and effective hemostasis is crucial as the first stage of wound healing. Some studies suggest that Na-CMC contributes to hemostasis probably through absorbing blood fluid leading to concentrated blood solids, activating the intrinsic coagulation pathway due to its negatively charged surface, and acting as a bridge for fibrin polymerization (Ohta et al., 2015). Its hemostatic activity can be further enhanced by incorporating Ca^2+^ ions (Kim et al., 2018). However, there are few reports regarding the hemostatic performance of HACC. In this study, to promote hemostatic activity, Ca^2+^ ions were incorporated into Na-CMC by partial substitution of original Na^+^ ions (Figure 2A), forming the Ca/Na-CMC layer of the multilayer films. The *in vitro* blood clotting test confirmed the enhanced hemostatic activity of Ca/Na-CMC layer compared to Na-CMC layer (Figure 5), which was consistent with previous studies (Chen et al., 2015; Kim et al., 2018; Xuan et al., 2020). Interestingly, although many studies report the hemostatic activity of chitosan regarding absorbing red blood cells and platelets due to its polycation feature (Gu et al., 2010), our results suggested that the capacity of HACC to accelerate blood clotting was not evident. Moreover, the hemostatic activity of Ca/Na-CMC was significantly reduced when homogeneous complexation occurred with HACC, which could be attributed to the reduction of water absorption capacity (Figure 4A) and change of surface charge responsible for activating the intrinsic coagulation pathway. In summary, Ca/Na-CMC layers could endow the multilayer films with promoted hemostatic activity owing to interfacial complexation resulting in separate Ca/Na-CMC and HACC layers.

Th risk of infection remains a significant issue before the epithelialization stage is accomplished. Plenty of efforts have been made to develop wound dressings with antibacterial capacity *via* involving antibacterial agents (e.g., silver, copper, various antibiotics, etc.) or modifying the dressing molecules with antibacterial structures (e.g., quaternary ammonium, quaternary phosphine, etc.) (Homaeigohar and Boccaccini, 2020; Rahimi et al., 2020). Silver is one of the most frequently used antibacterial agents due to its effective broad-spectrum activity, but its associated toxicity requires extremely precise control, which becomes its major limitation (Huang et al., 2020) (Shakya et al., 2019). Antibiotics are also effective in killing bacteria but suffering from a growing drug resistance, leading to more stringent use control (Homaeigohar and Boccaccini, 2020). Alternatively, HACC derived from the quaternization of chitosan seems to be a promising candidate for enhancing antibacterial activity, considering that chitosan is an approved wound dressing material (Jayakumar et al., 2011). In this study, HACC served as a separate layer (Figure 3B) responsible for antibacterial activity in the multilayer film. The antibacterial results confirm that HACC layers play a significant role for antibacterial activity, since the antibacterial rates increased with increasing the proportion of HACC layers (Figure 6). Remarkably, the antibacterial activity of HACC has been preserved and exploited more effectively when presented in the form of separate layers rather than homogeneous complexation with CMC. Considering the antibacterial mechanism of HACC, which involves the interaction of the positively charged quaternary ammonium groups with the negatively charged cytomembrane of bacteria, causing distorted membrane permeability, the positive charge density is essential for the antibacterial activity of HACC (Peng et al., 2010). However, it is well known that the complexation of oppositely charged polyelectrolytes, HACC and CMC in this study, can reduce the positive charge density of polycations (Schaaf and Schlenoff, 2015), leading to suppressed antibacterial activity. Therefore, the multilayer strategy limited the complexation to the interface between HACC and CMC, thus preserving the positive charge density of HACC and enhancing antibacterial activity.

Another concern about wound dressing is biocompatibility. Although HACC is considered a non-cytotoxic material, its cytotoxicity is still controversial depending on the degree of substitution, cell type, etc. (Peng et al., 2010; Zhou et al., 2014; Yang et al., 2016). In this study, L929 fibroblasts cell line was adopted to evaluate the films’ cytocompatibility, since fibroblasts play a crucial role throughout the wound healing process. The regular cell morphology and RGRs greater than 90% for all samples proved the lack of cytotoxicity. A clear tendency was found that both the RGRs and observed cell numbers from CLSM images decreased with increasing the content of HACC (Figure 7). Therefore, suggesting a mild negative effect of the HACC layer on cell proliferation. Fortunately, the Ca/Na-CMC layer presented a positive impact outstripping the negative effect of the HACC layer, as evidenced by the promoted cell proliferation in MF1 and MF2 groups compared to the control. Moreover, confirmed by the significantly higher RGRs in MF2 than in BF, despite of their same composition of HACC and Ca/Na-CMC, it can be concluded that the multilayer structures are superior to the homogeneous ones in promoting cell proliferation.

In summary, the fabrication of multilayer films *via* interfacial complexation offers a new strategy for combining polyanion and polycation. The strategy can not only effectively preserve and exert the biological function of each polyelectrolyte but also take advantage of the interfacial PEC layer, such as providing mechanical stability when swelling. Based on the *in vitro* results, MF2 showed excellent performance, and thus being evaluated for *in vivo* wound healing compared with BF. The *in vivo* results indicate that MF2 contributes to wound healing and exhibits the best wound healing efficacy compared to the others (Figure 8). It is perhaps due to the multilayer structure, which preserved each layer’s biological functions and exhibited superior physicochemical properties for wound healing. In the multilayer films, the Ca/Na-CMC layers are in direct contact with the wounds, and provide initial adhesion onto the injury, enhanced hemostatic effect due to Ca^2+^ and carboxymethyl groups, and promotion of cell proliferation; the HACC layers are faced with the external environment, and serve as active shields with continuous antibacterial function to prevent wound infection; the interfacial PEC layers separate the above two layers, and are essential for structural stability (Figures 5–7). In addition, the multilayer films possess excellent water absorption capability and adequate WVT for maintaining a moist environment and absorbing excessive exudate. In contrast, homogeneous complexation leads to reduced water absorption and WVT, which is unfavorable for removing excessive exudate duly.

## 5 Conclusion

We successfully fabricated a multilayer dressing composed of natural polyanions (Ca/Na-CMC) and polycations (HACC) based on interfacial complexation for multi-functionalization of wound dressings. The multilayer dressing composes of a Ca/Na-CMC layer for hemostasis and promotion of cell proliferation, a PEC layer formed at the interface for structural stability, and a HACC layer for antibiosis. Owing to interfacial complexation resulting in separate layers, the biological functions (hemostasis, antibiosis, etc) of Ca/Na-CMC and HACC are effectively preserved. Thus, the performances of each polyelectrolyte in the multilayer dressing are more predictable and active than in the blend dressing based on current homogeneous complexation. Consequently, the multilayer dressing shows higher hemostatic and antibacterial activity and promoted cell proliferation *in vitro*, compared to the blend dressing. Moreover, the multilayer dressing accelerated *in vivo* wound healing and promoted granulation tissue maturation. Our findings suggest the multilayer dressing a promising dressing candidate for effective wound management, and offer an alternative strategy to combine natural polyanions and polycations in the field of biomedical materials.

## Data Availability

The raw data supporting the conclusions of this article will be made available by the authors, without undue reservation.
